# Comparison of named entity recognition methodologies in biomedical documents

**DOI:** 10.1186/s12938-018-0573-6

**Published:** 2018-11-06

**Authors:** Hye-Jeong Song, Byeong-Cheol Jo, Chan-Young Park, Jong-Dae Kim, Yu-Seop Kim

**Affiliations:** 1 School of Software, Hallym University, Chuncheon, South Korea; 2 Bio-IT Research Center, Hallym University, Chuncheon, South Korea

**Keywords:** Biomedical named entity recognition (Bio NER), Recurrent neural network (RNN), Conditional random fields (CRFs), Word embedding

## Abstract

**Background:**

Biomedical named entity recognition (Bio-NER) is a fundamental task in handling biomedical text terms, such as RNA, protein, cell type, cell line, and DNA. Bio-NER is one of the most elementary and core tasks in biomedical knowledge discovery from texts. The system described here is developed by using the BioNLP/NLPBA 2004 shared task. Experiments are conducted on a training and evaluation set provided by the task organizers.

**Results:**

Our results show that, compared with a baseline having a 70.09% F1 score, the RNN Jordan- and Elman-type algorithms have F1 scores of approximately 60.53% and 58.80%, respectively. When we use CRF as a machine learning algorithm, CCA, GloVe, and Word2Vec have F1 scores of 72.73%, 72.74%, and 72.82%, respectively.

**Conclusions:**

By using the word embedding constructed through the unsupervised learning, the time and cost required to construct the learning data can be saved.

## Background

Named entity recognition (NER) assigns a named entity tag to a designated word by using rules and heuristics. The named entity, which presents a human, location, and an organization, should be recognized [1]. Named entity recognition is a task that extracts nominal and numeric information from a document and classifies the word into a person, an organization, or a date category [2]. NER classifies all words in the document into existing categories and “none-of-the-above”.

Biomedical named entity recognition is very important in language processing of biomedical texts, especially in extracting information of proteins and genes such as RNA or DNA from documents. Finding named entities of genes from texts is a very important and difficult task [3]. Finding a gene name in texts corresponds to finding a company name or a human name in newspapers. Recognizing biomedical named entities seems to be more difficult than recognizing normal named entities [4]. Numerous research studies have recognized named entities by using supervised learning algorithms based on many rules [5].

Supervised learning approaches have used Hidden Markov Models (HMMs) [6], decision trees [7], support vector machines (SVMs) [8], and conditional random fields (CRFs) [9, 10]. Supervised learning methods normally train with data of many features based on various linguistic rules, and evaluate the performance with test data that could not be found in the training data.

In this paper, we compare the performances of recurrent neural networks of deep learning with conditional random fields. A recurrent neural network (RNN) uses a Jordan-type algorithm and an Elman-type algorithm. We also measure the performance of conditional random fields using word embedding as their features. Word embedding has increased performance in natural language processing, machine translation, voice recognition, and so on [11]. Word embedding has been used as features in natural language processing and is mapped from a word in the higher-dimensional space into a real-numbered vector in the lower-dimensional space. Word2Vec, canonical correlation analysis (CCA), and global vector (GloVe) are used as word embedding methodologies in this paper. We compared two RNN algorithms and CRFs using three word embedding methods for named entity recognition in biomedical literature.

In the rest part of “Background”, we explain named entity recognition, particularly for biomedical texts. We introduce detailed methodologies and basic features used in this paper in “Methods”. “Results and discussion” shows the experimental results and evaluations, and “Conclusion” is our conclusion.

### Biomedical named entity recognition

Named entity recognition (NER) classifies all unregistered words appearing in texts and is a subtask of information extraction. Normally, NER uses eight categories—location, person, organization, date, time, percentage, monetary value, and “none-of-the-above” [12, 13]. NER first finds named entities in sentences and declares the category of the entity. In the sentence:

“Apple [**organization**] CEO Tim Cook [**Person**] Introduces 2 New, Lager iPhones, Smart Watch at Cupertino [**Location**] Flint Center [**Organization**] Event [14].”

“Apple” is recognized as an organization name instead of a fruit name in terms of its context. The words “Tim” and “Cook” are altogether recognized as a single word having a meaning of CEO of the Apple Company and a person’s name. “Cupertino” is a city name in California and is recognized as a location name, and “Flint” and “Center” are considered as a single name and recognized as an organization name.

Named entity recognition has three approaches—dictionary based, rule based, and machine learning based. A dictionary-based approach stores as many named entities as possible in a list called a gazetteer. This approach seems to be very simple, but at the same time has limitations. The NER is difficult because the target words are mainly proper nouns or unregistered words. In addition, new words can be generated frequently, and even the same word stream could be recognized as diverse named entities in terms of their current context [15, 16]. The second approach of the NER is a rule-based approach [17]. This approach ordinarily depends on the rules and patterns of named entities appearing in real sentences. Although rule-based approaches can use context to solve the problem of multiple named entities, every rule should be written before it is actually used. The third approach, the machine learning-based approach, tags the named entities to words even when the words are not listed in the dictionary and the context is not described in the rule set. For these approaches, support vector machines (SVMs) [18], Hidden Markov Models (HMMs) [6, 19], Maximum Entropy Markov Models (MEMMs) [20], and conditional random fields (CRFs) [9, 10] are mainly utilized.

Natural language processing researchers have been interested in the information extraction of genes, cancer, and protein from biomedical literature [21–24]. Biomedical named entity recognition, which is essential to biomedical information extraction, has been treated as the first stage of text mining in biomedical texts. For years, recognizing technical terms in the biomedical area has been one of the most challenging tasks in natural language processing related to biomedical research [25]. In this paper, we use five categories (protein, DNA, RNA, cell type, and cell line) instead of the categories used in the ordinary NER process. An example of the NER tagged sentence is as follows:

“IL-2 [**B-protein**] responsiveness requires three distinct elements [**B-DNA**] within the enhancer [**B-DNA**].”

Biomedical NER faces difficulties for five reasons. First, because of current researches, the number of new technical terms is rapidly increasing. It is very difficult to build a gazetteer that includes all of the new terms. Second, the same words or expressions could be classified as differently named entities in terms of their context. Third, the length of an entity is quite long, and the entity could include control characters such as hyphens (e.g., “12-*o*-tetradecanoylphorbol 13-acetate”). Fourth, abbreviation expressions are frequently used in the biomedical area, and they experience sense ambiguity. For example, “TCF” could refer to “T cell factor” or to “Tissue Culture Fluid” [26, 27]. Finally, in biomedical terms, normal terms or functional terms are combined, which is why a biomedical term can become too long. For example, “HTLV-I-infected” and “HTLV-I-transformed” include the normal terms “I”, “infected”, and “transformed”. It is difficult for biomedical NER to segment the sentence with named entities. Spelling changes also create a problem [28]. In addition, the named entity of one category could subsume another named entity of another category [29].

## Methods

We perform named entity recognition for words in a sentence by using CRFs and RNN, and compare the performance of each method. We use a BioNLP/NLPBA 2004 corpus [30, 31] of 22,402 sentences. We use 18,546 sentences as a training data set, and 3856 sentences as a test data set. The corpus are tagged with “protein”, “DNA”, “RNA”, “cell line”, and “cell type” categories. The next section describes CRFs and word embedding, and the rest explains RNN.

### Conditional random fields

A CRF is a statistical sequence modeling framework first introduced in [32]. CRFs are a class of statistical modeling methods often applied in pattern recognition and machine learning, where they are used for structure prediction. Whereas an ordinary classifier predicts a label for a single sample without regard to “neighboring” samples, a CRF can take context into account [33]. The reason why CRFs are more effective than HMMs is that CRFs use the conditional probability property instead of the independence assumption mainly used in HMMs. CRFs also avoid label bias problems and avoid the weaknesses of other Markov models derived from MEMMs and graphic models. CRFs show better performance than MEMMs and HMMs in bioinformatics, computational linguistics, and voice recognition. CRFs are also used for the prediction and analysis of labels for data in natural language writing. Features can be chosen randomly, and they are to be normalized to obtain solution [32, 34].

In this model, ${\text{X}} = \left\{ {\varvec{x}_{1} ,\varvec{x}_{2} ,\varvec{x}_{3} , \ldots  \varvec{x}_{T} } \right\}$ are the input data in which components are connected in sequence, and ${\text{Y}} = \left\{ {y_{1} ,y_{2} ,y_{3} , \ldots  y_{T} } \right\}$ are the labels for each component of the input data. In other words, when a new ***x*** is given, a *y* value is predicted using the following model:

1 $$ {\text{p}}\left( {{\text{y|}}{\mathbf{x}}} \right) = \frac{1}{{z\left( \varvec{x} \right)}}\mathop \prod \limits_{t = 1}^{T} { \exp }\left\{ {\mathop \sum \limits_{k = 1}^{k} \omega_{k} f_{k} \left( {y_{t} ,y_{t - 1} ,\varvec{x}_{t} } \right)} \right\} $$

2 $$ {\text{z}}\left( {\mathbf{x}} \right) = \mathop \sum \limits_{y} { \exp }\left( {\mathop \sum \limits_{k} \omega_{k} f_{k} \left( {y,\varvec{x}} \right)} \right), $$

where z(x) standardizes the probability value, and $f_{k}$ is a feature function, which is a characteristic function on feature *k*. This function returns 1 when the given input ${\text{y}}_{\text{t}} ,y_{t - 1} ,\varvec{x}_{t}$ includes a feature *k,* and returns 0 otherwise. $\omega_{k}$ is the weight of the feature. In this study, a CRF suite [35] was used to make predictions by using the average perceptron generated by the CRF algorithm.

### Word embedding

**Fig. 1:**
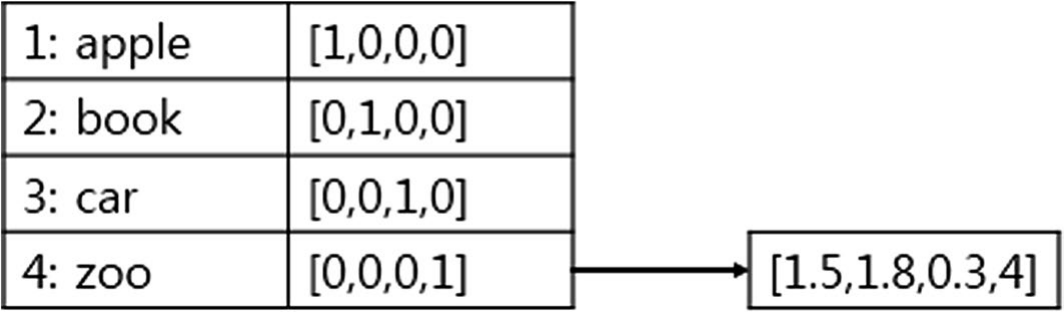
One-hot vs. word embedding. The left vector inside a table is one-hot representation, and the right vector inside a small rectangle is word embedding representation

Word embedding is also called word representation or distributed representation. It learns vector representation for every word appearing in the corpus. Previous research studies represented a word as a one-hot representation. The one-hot representation uses a vocabulary-sized vector, and takes a 1 when the word appears in the document and 0 when it does not [36]. Word embedding reduces the dimensions and sparseness of the original vector and fills the vector with real numbers. Figure 1 shows the difference between one-hot representation and word embedding.

### Word2Vec

**Fig. 2:**
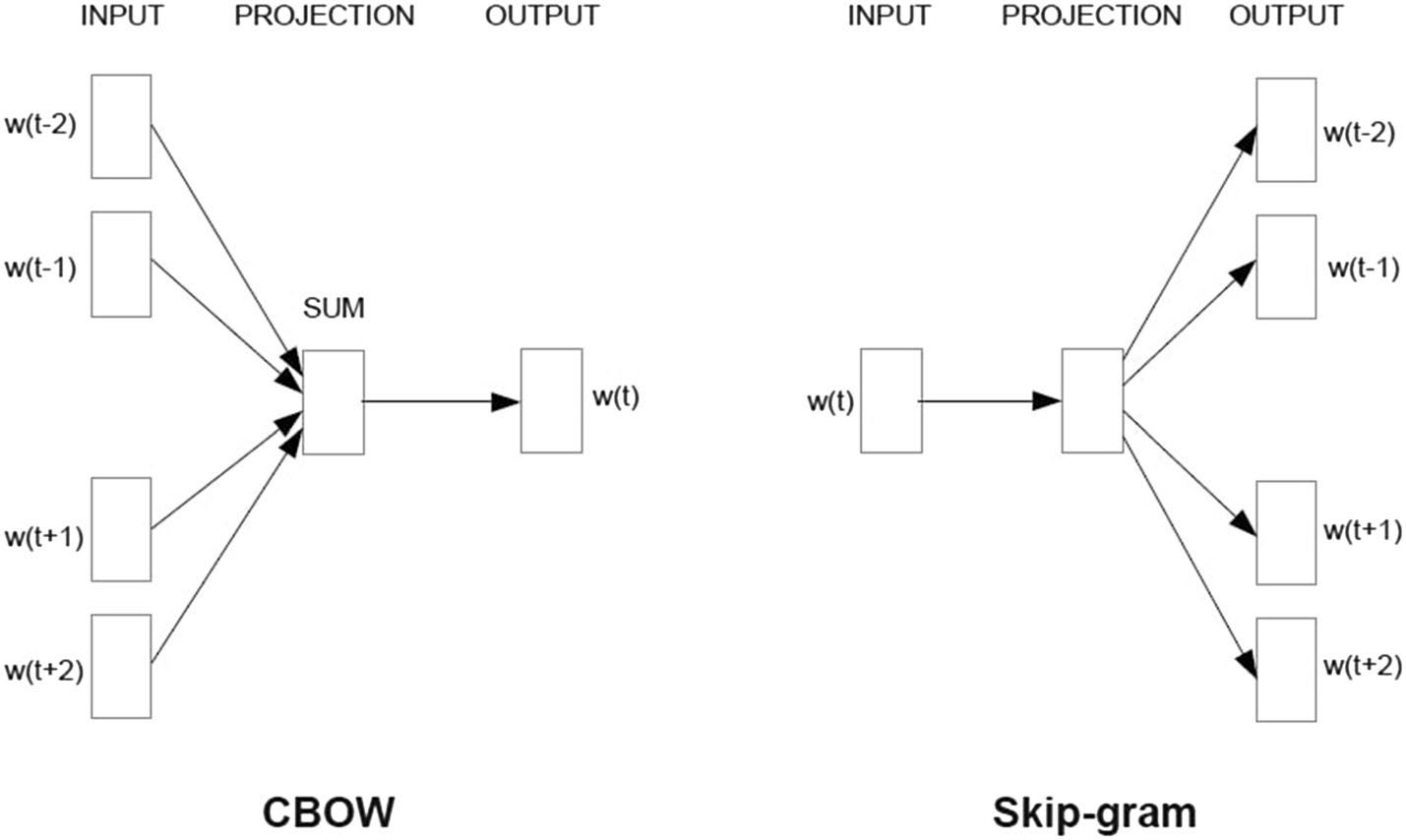
Word2Vec architecture

Word2Vec assumes that the words sharing the same context could have similar meanings. It classifies words near to the given word into related words and learns the words using artificial neural networks. Word2Vec has two structures: Continuous Bag of Words (CBOW) and skip gram architectures. Figure 2 shows the Word2Vec architecture [37].

The CBOW side has surrounding words w(t − 2), w(t − 1), w(t + 1), and w(t + 2) as input, and predicts w(t) as output. The skip-gram side uses w(t) as its input and predicts w(t − 2), w(t − 1), w(t + 1), and w(t + 2) as output.

### Global vector (GloVe)

GloVe is an unsupervised learning algorithm for obtaining vector representations for words. Training is performed on aggregated global word-word co-occurrence statistics from a corpus, and the resulting representations showcase interesting linear substructures of the word vector space [38]. GloVe considers the global context as well as the local context [39].

3 $$ \mathop \sum \limits_{i,j = 1}^{v} f\left( {x_{ij} } \right)({\text{e}}{}_{i}^{T} \widetilde{e}_{j} + y_{i} + \widetilde{y}_{j} - \log X_{ij} )^{2} , $$

where *X* is a word co-occurring in a matrix, *X*_*ij*_ is the frequency of the co-occurrence of word *i* and word *j*, and $X_{i} = \mathop \sum \nolimits_{k}^{v} X_{ik}$ is the total number of occurrences of word *i* in the corpus. The occurrence probability of a word *j* in the context of a word *i* is $X_{ij} = P\left( {j |i} \right) = X_{ij} /x_{i}$. e is word embedding, and $\widetilde{e}$ is a separate-context word embedding. ${\text{f}}\left( {X_{ij} } \right)$ indicates the weight and has three conditions. First, *f(0)* = 0. Second, *f(x)* does not decrease not to give weights to very rarely co-occurring words. Third, *f(x)* should be relatively smaller than the large value of *x* so it does not give weight to frequently co-occurred words.

### Canonical correlation analysis (CCA)

Canonical correlation analysis (CCA) was introduced by Hotelling [40]. CCA is a statistical method to investigate the relationship between two variable sets, and it can concurrently examine the correlation of variables belonging to different sets. CCA finds correlations between two variable sets (*X, Y*), and also finds parameters that maximize the correlation coefficients [41]. CCA can be calculated directly from the data set, and can also be calculated after transforming the data sets into covariance matrices. These two methods are represented based on singular value decomposition. In [42, 43], if CCA is used to predict labels in data, string theory guarantees the correspondence to lower-dimensional embedding. CCA tries to find two projection vectors to maximize the correlation. Using random variables (*X, Y* $\in$ *R*), where *X* is a word representation and *Y* is its related context representation, CCA tries to find *k*-dimensional projection vectors that maximize the correlation between two variables [44].

Assuming that we have two variables $x \in C^{{d_{1} }} , \quad y \in C^{{d_{2} }}$, CCA can be defined as a problem to maximize the correlation between two variables on *X* and *Y* vectors. With a pair of vectors ${\text{x}} = \hat{w}_{x}^{T} x,\quad  {\text{y}} = \hat{w}_{\text{y}}^{T} y$, we can use the following correlation expression:

4 $$ {\text{p}} = \frac{{{\text{E}}\left[ {\text{xy}} \right]}}{{\sqrt {\left[ {x^{2} } \right]E\left[ {y^{2} } \right]} }} = \frac{{w_{x}^{T} C_{xy} w_{y} }}{{\sqrt {w_{x}^{T} C_{xx} w_{x} w_{y}^{2} C_{yy} w_{y} } }} $$

where $C_{xy} = E\left[ {xy^{T} } \right], \;C_{xx} = E\left[ {xx^{T} } \right], \;{\text{and}}\; C_{yy} = E\left[ {yy^{T} } \right]$. The first eigenvectors $\hat{w}_{{x_{1} }} , \hat{w}_{{y_{1} }}$ can be the first correlation *P*_*1*_, and the second eigenvectors can be the second correlation *P*_*2*_ [45].

### Recurrent neural network

In machine learning and cognitive science, artificial neural networks (ANNs) are a family of models inspired by biological neural networks that are used to estimate or approximate functions that can depend on a large number of inputs and are generally unknown [46]. ANNs work well in nonlinear functions and pattern recognition. Many researchers working in data mining, artificial intelligence, and bioinformatics have been interested in ANNs for its diverse applications [47].

**Fig. 3:**
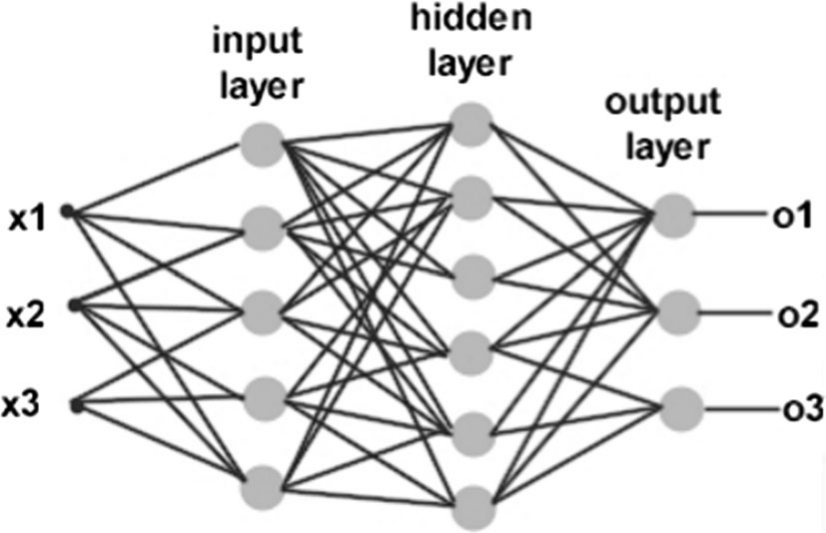
Simple neural network. This network takes *x* values as its inputs and makes *o* values as its outputs. This network is composed of an input layer, a hidden layer, and an output layer

Figure 3 [48] shows a simple ANN structure. ANNs use an activation function with a combination function of input variables and input values. The input layer takes input values for its training, and the hidden layer is located between the input layer and the output layer. Training is performed mainly in the hidden layer and tries to find the optimum weight value set labeled on each edge. A sigmoid function is used on each node to calculate each node’s output after summing its inputs.

A recurrent neural network (RNN) has connections between nodes to form a directed cycle. Unlike normal feedforward networks, RNN can also use feedback systems [49]. RNN has shown outstanding performance in various natural-language processing tasks. The basic idea of RNN comes from the mechanism of sequential labeling. Normal ANNs assume independence between their inputs and outputs. RNN applies the same tasks to every component of the sequence, and the output is affected by the previous calculation results. In other words, the network is designed so that input *x*_*t*_ of time *t* and the previous hidden layer’s output of time *t* − 1 can contribute to the hidden layer’s output of time *t*. Although RNN can be applied to any sequence length, shorter sequences show better performance [50].

**Fig. 4:**
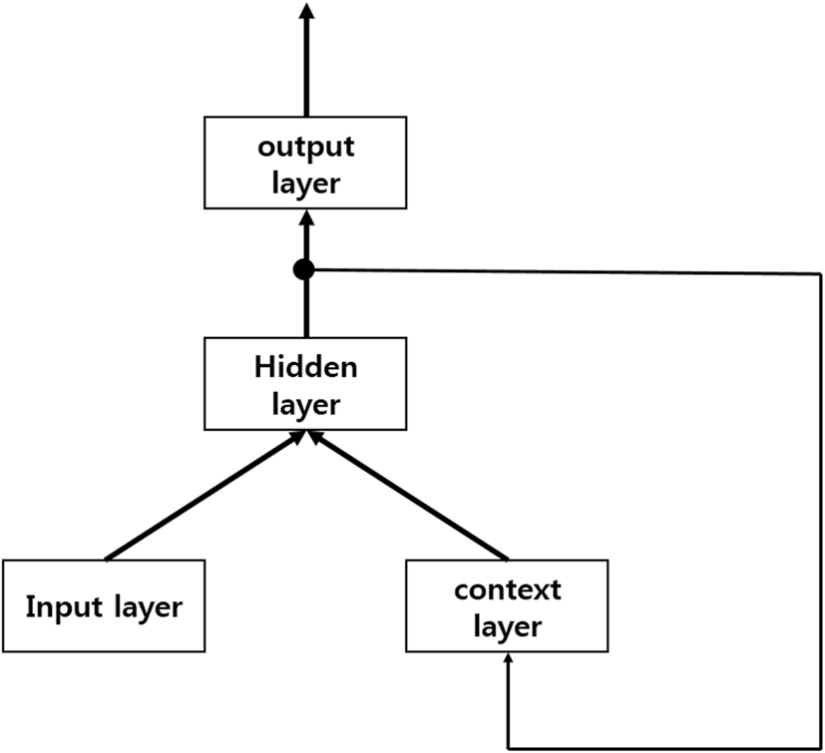
Elman-type network. Output of hidden layer goes to input of context layer

We apply an RNN algorithm by using an RNN tutorial [51]. RNN has two types: the Elman-type network [52] and the Jordan-type network [53]. The Elman-type network adds a context layer to the normal RNN and feeds back the hidden layer’s output to the context layer’s input. This network feeds back the output value to the hidden layer rather than the input layer. The hidden layer of this network plays the same role as the input layer of a normal RNN. Figure 4 shows the basic structure of the Elman-type RNN. The output of the hidden layer, a sigmoid function of each node, and the output value of this network are explained below:

5 $$ h\left( t \right) = f\left( {Ux\left( t \right) + Vh\left( {t - 1} \right)} \right) $$

6 $$ {\text{f}}(x) = \frac{1}{{1 + {\text{e}}^{{ - {\text{x}}}} }} $$

7 $$ {\text{y}}_{\text{t}} = g\left( {Wh\left( t \right)} \right) $$

In (5), which shows the output of the hidden node, *U* is a matrix of raw input values and current hidden nodes, and *V* is a matrix of the context node and the previous hidden node. Expression (6) shows a sigmoid function, and (7) shows the output value.

**Fig. 5:**
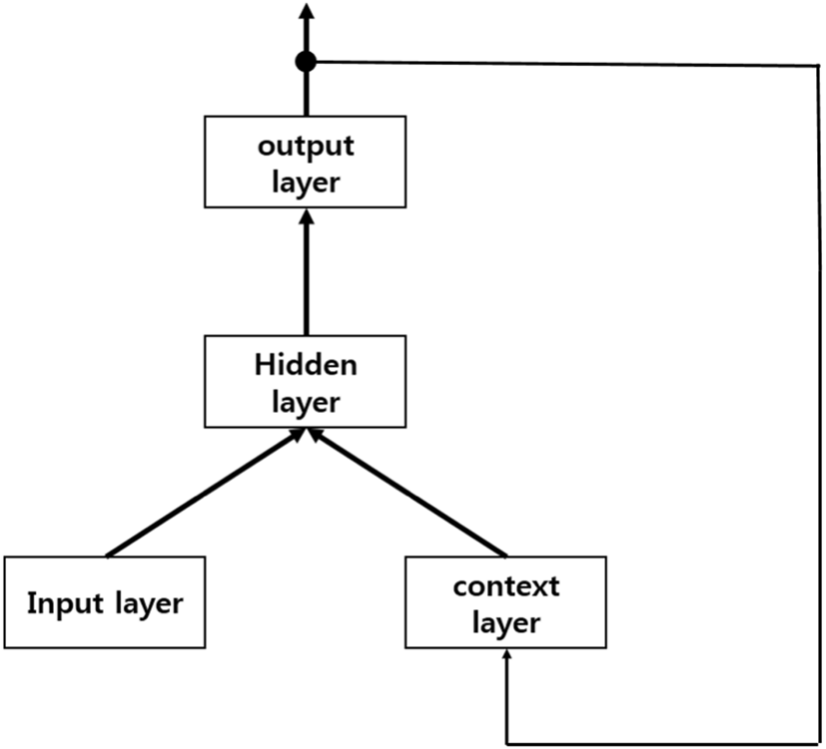
Jordan-type network. Feedback starts from output layer, not the hidden layer

The Jordan-type network shown in Fig. 5 is very similar to the Elman-type network, except that the feedback is coming from the output layer rather than the hidden layer. The hidden layer’s output is calculated by the following expression:

8 $$ h\left( t \right) = f\left( {Ux\left( t \right) + Vy\left( {t - 1} \right)} \right) $$

We use negative log-likelihood as a loss function. The gradient descent uses the mini-batch gradient descent method. This method does not apply the gradient descent method to each data, but calculates the gradient by batch and reflects it to the next learning. We apply a mini-batch gradient descent to one batch in one sentence. Because the length of the sentences in the corpus are all different, this method works well.

### Feature

This study uses n-Gram features for a baseline experiment of the conditional random fields. Recurrent neural networks use raw word sequences for their inputs. Table 1 lists the unigrams, bigrams, and trigrams used in this study.

**Table 1:** N-Gram description

Feature	Description
Unigram	${\text{w}}_{{{\text{i}} - 2}} ,{\text{w}}_{{{\text{i}} - 1}} ,{\text{w}}_{\text{i}} ,{\text{w}}_{{{\text{i}} + 1}} ,{\text{w}}_{{{\text{i}} + 2}}$
Bigram	${\text{w}}_{{{\text{i}} - 2}} \left| {{\text{w}}_{{{\text{i}} - 1}} ,{\text{w}}_{{{\text{i}} - 1}} } \right|{\text{w}}_{\text{i}} ,{\text{w}}_{\text{i}} \left| {{\text{w}}_{{{\text{i}} + 1}} ,{\text{w}}_{{{\text{i}} + 1}} } \right|{\text{w}}_{{{\text{i}} + 2}}$
Trigram	${\text{w}}_{{{\text{i}} - 2}} \left| {{\text{w}}_{{{\text{i}} - 1}} } \right|{\text{w}}_{\text{i}} ,{\text{w}}_{{{\text{i}} - 1}} \left| {{\text{w}}_{\text{i}} } \right|{\text{w}}_{{{\text{i}} + 1}} ,{\text{w}}_{\text{i}} \left| {{\text{w}}_{{{\text{i}} + 1}} } \right|{\text{w}}_{{{\text{i}} + 2}}$

For the sentence, “Tumor and serum beta-2-microglobulin expression in women with breast cancer”, let us assume that w_i_ is “breast”. Then, w_i−2_, w_i−1_, w_i+1_ and w_i+2_ are “women”, “with”, “cancer” and “.”, respectively. w_i−1_|w_i_ of the bigram is “with|breast”, and w_i−2_|w_i−1_|w_i_ is “with|breast|cancer”.

## Results and discussion

We use a BioNLP/NLPBA 2004 shared corpus for the experiment. In this experiment, we compare the performance of RNN and CRFs with word embedding. For the baseline, only n-Gram (unigram, bigram, trigram) features of CRFs are utilized. The Jordan-type RNN and Elman-type RNN are compared, and at the same time, Word2Vec, GloVe, and CCA of the CRFs are also compared. For performance evaluation, we set the word embedding dimension to 100, the window size to 5, the number of hidden units to 100, and the number of hidden layers to 1.

We use the F1 score as the performance measurement. The F1 score is calculated by the following expression:

9 $$ {\text{F}}1\;{\text{score}} = \frac{{2 * {\text{precision*recall}}}}{{{\text{precision}} + {\text{recall}}}}, $$

where the precision is a ratio of true positives from the positive side, and recall is a ratio of true positives from the true side.

In this experiment, the Jordan-type RNN shows an F1 value of 60.75%, and the Elman-type RNN has an F1 value of 58.80%. For the CRFs’ performance measurement, we apply various dimensions of word embedding (10, 30, 50, 80, 100), window sizes (3, 5, 7, 9, 11), and the minimum frequency (3).

**Fig. 6:**
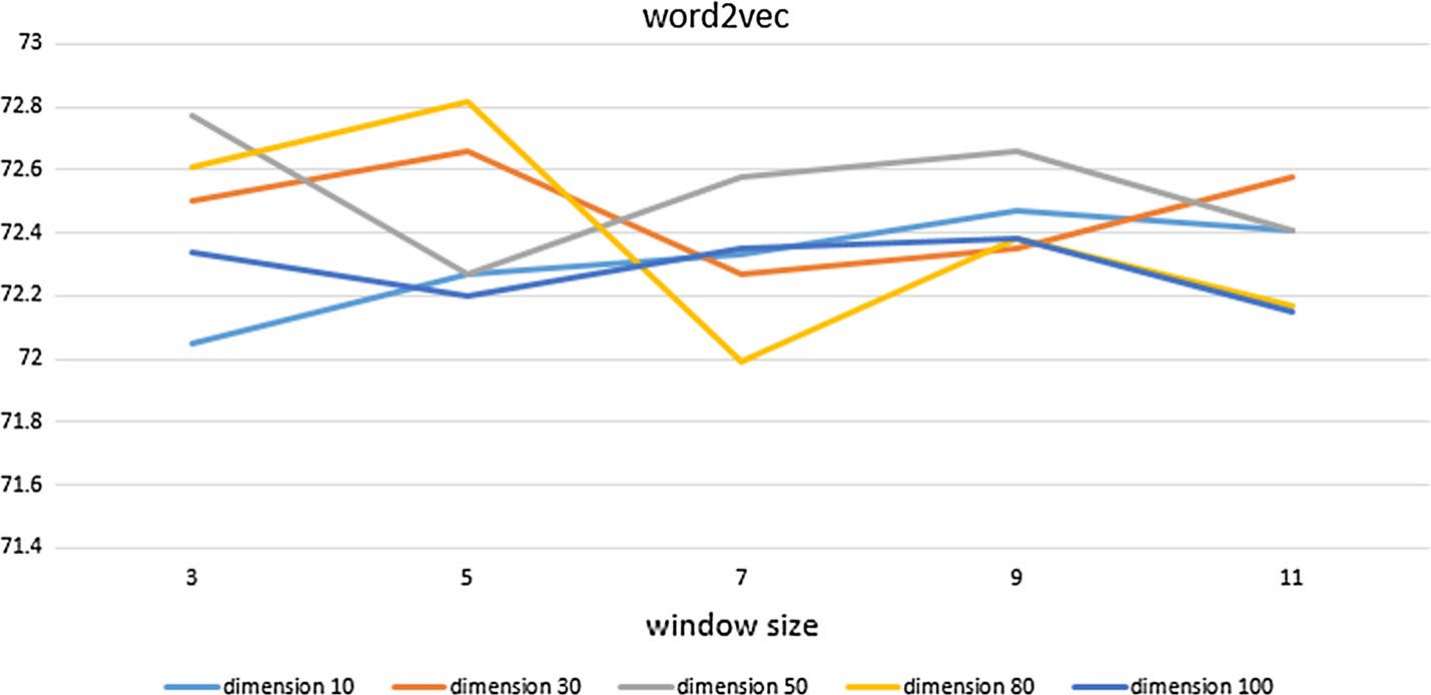
Experimental results of Word2Vec in CRFs. X-axis indicates window size, and Y-axis indicates performance. Five lines correspond to five dimension sizes

**Fig. 7:**
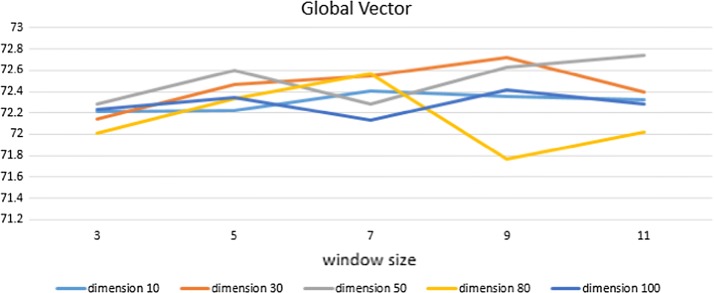
Experimental results of Global Vector in CRFs. The X-axis indicates window size, and Y-axis indicates performance. Five lines correspond to five dimension sizes

**Fig. 8:**
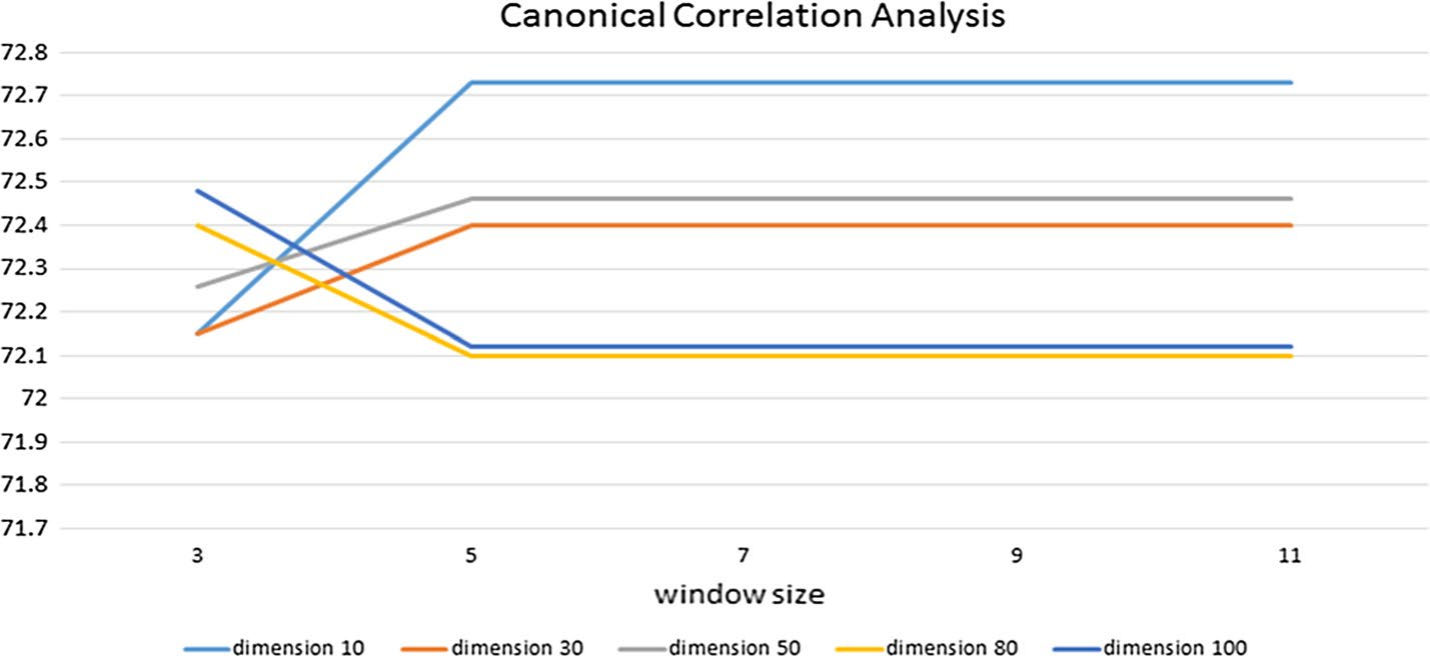
Experimental results of CCA in CRFs. X-axis indicates window size, and Y-axis indicates performance. Five lines correspond to five dimension sizes

Figures 6, 7 and 8 show the experimental results of each word embedding method with various dimensions and window sizes.

Word2Vec shows the highest performance when the dimension size is 80 and the window size is 5. However, the same line shows the lowest performance when the window size is changed to 7. In Fig. 6, the line with dimension size of 50 shows a relatively stable and high performance for all window sizes. Word2Vec does not seem to need high-dimensional representation, and lower-dimensional representations show an increase in performance proportional to the window size. Higher-dimensional representations do not exhibit particular characteristics in Word2Vec.

In GloVe, a representation of 50 dimensions and 11 window sizes shows the highest performance. Like Word2Vec, GloVe also shows relatively stable and high performance when its size of dimension is 50. Of course, the 30-dimensional representation also shows a good result.

Figure 8 shows that lower-dimensional cases have relatively higher performance than higher-dimensional cases when CCA is used for word embedding.

Table 2 lists the results compared with well-known former research results. Our system shows an F1 score of up to 72.82%, which is the highest of all the results in Table 2, when CRFs of Word2Vec are used. Zhou et al. [54] used HMM and SVM to achieve 72.55% for the BioNLP/NLPBA shared task 2004, and their achievement has been the highest until now. At the same competition, Song et al. achieved 66.28% by using HMM and CRF. Ponomareva et al. [55] used HMM and achieved 65.7%, and Saha et al. [29] used Maximum Entropy to obtain an F1 score of 67.41%. Findel et al. [56], Settles [57], and Tsai et al. [58] reported scores of 69.8% to 70.2%, which could not overcome the results from Zhou and Su. Our system shows a maximum score of 72.82%, which is approximately 0.3% points higher than Zhou and Su’s scores when using Word2Vec-based CRFs. Word embedding is also advantageous in that it is automatically constructed through the unsupervised learning, while the existing methodology uses data that is directly labeled by a person. Our approach does not require any domain knowledge, a dictionary, or other outside resources, but we were able to show the highest performance of all tested methods.

**Table 2:** Performance comparison by using BioNLP/NLPBA 2004 corpus

System	Methodology	F1 score (%)
Our system	CRF	
	Base line	71.09
	Word2vec	72.82
	Glove	72.74
	CCA	72.73
	RNN	
	Jordan	60.75
	Elman	58.80
Zhou and Su [54]	HMM, SVM	72.55
Song et al. [9]	SVM, CRF	66.28
Ponomareva et al. [55]	HMM	65.7
Saha et al. [29]	Maximum entropy	67.41

## Conclusion

Bio-NER has more difficulties than normal NER because technical terms in biomedical texts have unusual characteristics. We compared various machine-learning approaches based on CRFs and RNN. In this research, RNN exhibited a lower performance than CRFs. The disadvantage of RNN is that it does not remember old information. Also, since we did not find the optimal activation function and initialization method, RNN has lower performance than CRFs. We use a single hidden layer. However, RNN could be a very useful method in Bio-NER because of its unsupervised learning property. From an experiment, our method shows the highest performance of all the other experiments.

For the future study, our research will proceed in three directions. First, we will design a more optimized deep artificial neural network structure for the Bio-NER. Because we had limited knowledge and experience in deep artificial neural network, this study used a relatively simple model. Therefore, we will develop deep artificial neural network specialized on this problem based on accumulated knowledge and technology. Second, we would like to develop unsupervised learning methods for the Bio-NER. The lack of an annotated corpus is a barrier to new research. Although it has unsupervised learning properties, RNN requires an annotated corpus. We should develop fully- or semi-supervised learning methods for Bio-NER. Third, various linguistic resources for domain knowledge should be built for performance development. Gazetteers, word embedding methods, and other resources should be developed.

## Abbreviation

NER: named entity recognition
RNN: recurrent neural network
CRF: conditional random fields
CCA: canonical correlation analysis
GloVe: global vector
SVM: support vector machines
HMM: Hidden Markov Models
MEMM: Maximum Entropy Markov Models
NLP: natural language processing
